# Delivery of Fall Prevention Interventions for At-Risk Older Adults in Rural Areas: Findings from a National Dissemination

**DOI:** 10.3390/ijerph15122798

**Published:** 2018-12-10

**Authors:** Matthew Lee Smith, Samuel D. Towne, Angelica Herrera-Venson, Kathleen Cameron, Scott A. Horel, Marcia G. Ory, Chelsea L. Gilchrist, Ellen C. Schneider, Casey DiCocco, Shannon Skowronski

**Affiliations:** 1 Center for Population Health and Aging, Texas A&M University, College Station, TX 77843, USA; samuel.towne@ucf.edu (S.D.T.J.); mory@sph.tamhsc.edu (M.G.O.); 2 School of Public Health, Texas A&M University, College Station, TX 77843, USA; sahorel@sph.tamhsc.edu; 3 College of Public Health, The University of Georgia, Athens, GA 30602, USA; 4 Department of Health Management and Informatics, University of Central Florida, Orlando, FL 32816, USA; 5 Disability, Aging, and Technology Cluster, University of Central Florida, Orlando, FL 32816, USA; 6 Southwest Rural Health Research Center, Texas A&M University, College Station, TX 77843, USA; 7 National Council on Aging, Arlington, VA 22202, USA; angelica.herrera-venson@ncoa.org (A.H.-V.); kathleen.cameron@ncoa.org (K.C.); chelsea.gilchrist@ncoa.org (C.L.G.); ecschnei@email.unc.edu (E.C.S.); 8 Center for Aging and Health, School of Medicine, University of North Carolina at Chapel Hill, Chapel Hill, NC 27599, USA; 9 Administration for Community Living, Washington, DC 20201, USA; casey.dicocco@acl.hhs.gov (C.D.); shannon.skowronski@acl.hhs.gov (S.S.)

**Keywords:** rural, fall prevention, evidence-based program, dissemination, geospatial research

## Abstract

Falls incidence rates and associated injuries are projected to increase among rural-dwelling older adults, which highlights the need for effective interventions to prevent falls and manage fall-related risks. The purpose of this descriptive study was to identify the geospatial dissemination of eight evidence-based fall prevention programs (e.g., A Matter of Balance, Stepping On, Tai Chi, Otago Exercise Program) across the United States (U.S.) in terms of participants enrolled, workshops delivered, and geospatial reach. These dissemination characteristics were compared across three rurality designations (i.e., metro areas; non-metro areas adjacent to metro areas; and, non-metro areas not adjacent to metro areas). Data were analyzed from a national repository of 39 Administration for Community Living (ACL) grantees from 2014–2017 (spanning 22 states). Descriptive statistics were used to assess program reach, delivery-site type, and completion rate by rurality. Geographic information systems (GIS) geospatially represented the collective reach of the eight interventions. Of the 45,812 participants who attended a fall prevention program, 12.7% attended workshops in non-metro adjacent areas and 6.6% attended workshops in non-metro non-adjacent areas. Of the 3755 workshops delivered (in over 550 unique counties), most were delivered in senior centers (26%), residential facilities (20%), healthcare organizations (13%), and faith-based organizations (9%). On average, the workshop attendance/retention rates were consistent across rurality (~70%). Findings highlight the need to diversify the delivery infrastructure for fall prevention programs to adequately serve older adults in rural areas. Ongoing efforts are needed to offer sustainable technical assistance and to develop scalable clinical-community referral systems to increase fall prevention program participation among rural-dwelling older adults.

## 1. Introduction

Improving access to and utilization of interventions that prevent or delay potentially preventable falls is critical. With approximately one-in-four older adults aged 65 years and older falling each year [1], non-fatal and fatal injuries contribute to high rates of potentially preventable medical encounters and associated costs [2,3,4]. Although not all falls lead to significant injury and related hospitalization, those that do can be costly [5]. Further, even among community-dwelling adults, discharge after fall-related hospitalization can lead to additional care needs in institutionalized settings [6]. Fall-related incidence and related consequences are anticipated to rise as the older adult population, who are at a greater risk of falling, is growing. For example, those that are aged 65 years and older living in the United States (U.S.) increased from 35.0 million in 2000 (12.4% of the overall population) to 49.2 million in 2016 (15.2% of the overall population), and it is estimated that older adults will comprise approximately one-in-five persons in the U.S. by 2030 [7]. For these reasons, increasing the availability, accessibility, and affordability of fall prevention strategies is timely and of interest to multiple stakeholders (e.g., policy-makers, aging service organizations, older adults themselves and their families) [8]. One such set of strategies are evidence-based fall prevention interventions, which have been shown to successfully reduce falls, diminish fall-related risk factors, improve physical functioning, and improve quality of life [9].

There is no single solution to combat falls among older adults. The known risk factors are multi-factorial and they can be associated with biological, psychosocial, and environmental causes [10,11]. One extrinsic risk factor for falls is residing in rural areas. Rural-dwelling older adults are at an increased risk for falls relative to their urban-dwelling counterparts [12] because of differences in the physical environment and lifestyle activities [13]. Additionally, older adults in rural areas face social issues that are associated with geographic and technological isolation [14,15] and geospatially dispersed health-related services and resources [16,17]. Therefore, in the event of a fall, rural-dwelling older adults may experience longer wait times before emergency medical personnel arrive on the scene and longer travel times to reach advanced healthcare settings [18]. Beyond diminished access to traditional healthcare services, there are fewer health promotion programs that are available in rural areas [19], which limit protective and preventative service use among rural-residing older adults. These place-based issues may exacerbate the fall-related consequences for falls that may otherwise be less severe.

While healthcare access issues among older adults are especially pronounced in rural areas [20], the vast majority of non-clinical fall prevention activities occur outside formal healthcare settings. With an emphasis on fall prevention and management, a series of evidence-based fall prevention programs have been embedded for delivery in diverse community settings to assist older adults successfully age-in-place [21]. A growing infrastructure of trained health professionals and lay leaders has been established to implement evidence-based fall prevention interventions utilizing the aging services network, public health sector, and healthcare system [8]. Because multi-factorial solutions are needed to address multi-factorial problems, these evidence-based fall prevention programs often incorporate educational components (e.g., to reduce fear of falling and improve self-efficacy) and physical activity (i.e., to improve lower body strength, balance, and flexibility) [9]. Acknowledging that there is no one-size-fits-all approach to fall prevention programming, each intervention was specifically designed for an intended audience with differing health and functional needs [21,22].

Little is known about the simultaneous grand-scale dissemination of multiple evidence-based fall prevention programs and their reach to rural areas. Although some studies have examined the delivery of fall prevention interventions and their effectiveness in rural communities [23,24], these studies often focus on only one fall prevention intervention [25,26] or a limited geographic area [27]. Additional efforts are needed to assess the delivery of fall prevention programming in rural areas to determine their availability in high-risk communities as well as to inform strategic planning efforts to enhance the dissemination of effective interventions [28].

Utilizing a methodology published previously [29], the primary purpose of this descriptive study was to identify the geospatial dissemination of fall prevention programs across the U.S. in terms of total number of participants enrolled, workshops delivered, and counties reached. We compared the dissemination characteristics across rurality. Secondarily, data were also stratified by fall program and delivery site type to provide additional context about participant and workshop characteristics. 

## 2. Materials and Methods

### 2.1. Study Participants and Procedures

Data for this study utilized a national repository created alongside a series of funding initiatives to support the dissemination and sustainability of chronic disease self-management education (CDSME) and fall prevention programs across the U.S. [30,31]. The process for selecting standardized measures as well as data collection tools, coordination, and processes used to develop and operate this national, online database were similar to those that were used for CDSME programs [32]. Data components in the data repository include workshop information, participant information, workshop attendance records, and organization data for host and delivery sites. Workshop leaders and organizations hosting programs collect these data locally. Data can be entered in a centralized or de-centralized manner at the state or regional level. Although outcome data were collected at baseline and post-intervention, these data are not presented as part of this study. To select the outcomes used in this national initiative, a panel of experts was convened to identify and provide recommendations about the most relevant items that are associated with fall-related risk and measures capable of detecting participant improvement over the course of these fall prevention workshops. Members of this national panel represented the federal government, academic institutions, community organizations, and program developers. Data used for this study included efforts from 39 grantees (36 unique grantees with 3 funded more than once) spanning 22 states from 2014 to 2017. The national database is limited to data for the evidence-based fall prevention programs that were chosen by organizations (for a specific target number of participants) under their grant funded by the Administration for Community Living (ACL) through the Prevention and Public Health Fund (PPHF). Grantees are required to enter their data into this national data repository. These grantees’ efforts do not represent all Title III-D approved evidence-based fall prevention programs delivered by the network of aging and healthcare organizations across the U.S., which may be funded by the Older Americans Act Title III-D, the Centers for Disease Control and Prevention Arthritis Program, or other local, state, and private funding sources. Institutional Review Board approval was granted by The University of Georgia (#00000249) for this secondary, de-identified data analysis.

### 2.2. Data and Measures

#### 2.2.1. Delivery Site Rurality

Rural-Urban Continuum Codes (RUCC) were used to measure rurality at the county-level for organizations delivering the fall prevention program. RUCCs were developed by the U.S. Department of Agriculture and use a nine-point scale to indicate proximity to nearby metro areas [33,34,35]. For this study, three categories were created to document rurality in terms of adjacency to metro areas: metro areas (i.e., RUCC of 1, 2, and 3); non-metro adjacent areas (i.e., RUCC of 4, 6, and 8); and, non-metro non-adjacent areas (i.e., RUCC of 5, 7, and 9). More information about each of the nine RUCC classifications and associated methodology can be found elsewhere [33]. For the purposes of this study, rural areas are defined as non-metro areas (i.e., non-metro adjacent and non-metro non-adjacent). However, rather than using a simple rural-urban dichotomy, non-metro areas were further separated based on their distance to metropolitan areas (i.e., metro adjacency), because metro adjacency may affect access to care [36] or transportation options to program delivery sites.

#### 2.2.2. Workshop and County Characteristics

Using data in the national repository, the total number of participants enrolled, workshops delivered, and unique counties reached were tabulated. Based on the ZIP Code of the workshop delivery location, the ZIP Code Tabulation Area (ZCTA) was identified, and population statistics were obtained. More specifically, the ZCTA of the workshop delivery location was used to identify the median household income (i.e., 2011 inflation-adjusted dollars), percent of residents residing in poverty (i.e., ratio of income to poverty level in the past 12 months), percent of residents who were white, percent of residents who were Hispanic, percent of participants who were African American, and percent of residents with less than a high school education. The average number of participants enrolled in each workshop was calculated. Because each fall prevention program offers a different number of workshop sessions, the average proportion of workshop sessions attended was reported (i.e., ranging from 0% to 100%). 

#### 2.2.3. Program Type

Because this national initiative included multiple fall prevention programs, the dissemination of each unique workshop type was of interest. The eight programs included: A Matter of Balance (AMOB) [37]; Stepping On (SO) [38]; Tai Ji Quan: Moving for Better Balance (TJQMBB) [39]; Tai Chi for Arthritis (TCA) [40]; FallScape [41]; Stay Active and Independent for Life (SAIL) [41]; Stay Safe, Stay Active [42]; and, Otago Exercise Program (OEP) [43].

#### 2.2.4. Delivery Site Type

Given the diverse organizational infrastructure that was used to deliver fall prevention programs, the delivery site type for workshops was of interest. Delivery site types included: senior centers; residential facilities; healthcare organizations; faith-based organizations; recreational organizations; community centers; multi-purpose and social service organizations; libraries; Area Agencies on Aging; municipal governments; county health departments; educational institutions; tribal centers; state units on aging; workplaces; state departments of health; and, other site types [44,45].

#### 2.2.5. Sociodemographics

Personal characteristics of the participants that were enrolled in fall prevention programs were collected using self-reported survey instruments and included age, gender, race, ethnicity, education, and the most prevalent chronic conditions (e.g., arthritis, heart disease, diabetes).

### 2.3. Statistical Methods

All analyses were performed using SPSS (version 25, IBM Corporation, Armonk, NY, USA) for this descriptive study. Counts were tabulated for the number of participants enrolled, the number of workshops delivered, and the number of unique counties reached. These counts were stratified by workshop type and rurality (see Table 1). Counts were also stratified by delivery site type and rurality (see Table 2). Averages were calculated for participant characteristics, workshop delivery location characteristics (yielded from ZCTA of the workshop location), and workshop characteristics (see Table 3). ArcGIS (Environmental Systems Research Institute, Redlands, CA, USA) was used to geospatially map the delivery location of each workshop.

## 3. Results

**Figure 1:**
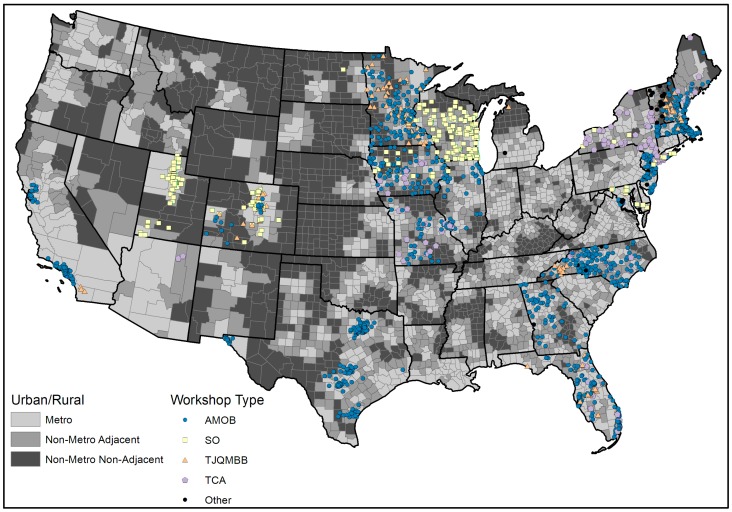
Geospatial distribution of fall prevention program workshops by county and rurality (2014–2017).

Figure 1 illustrates the national delivery of fall prevention programs by rurality. Darker shading indicates counties that are classified as more rural. For mapping purposes, four programs were combined to form an “other” category based on their smaller frequencies of workshops delivered. The blue (AMOB), yellow (SO), orange (TJQMBB), purple (TCA), and black (Other) shapes indicate the physical location of fall prevention program delivery sites (see Figure 1). The data used to generate Figure 1 corresponds with the data reported in Table 1 (although all eight programs are listed separately in Table 1). Table 1 also describes the delivery of individual fall prevention programs by rurality in terms of the number of participants enrolled, the number of workshops delivered, and the number of counties reached.

Overall, 45,812 participants were enrolled in fall prevention programs over the study period (2014–2017). The majority of participants attended workshops in metro areas (80.7%), followed by workshops in non-metro adjacent areas (12.7%), and non-metro non-adjacent areas (6.6%). Overall, 3755 workshops were delivered over the study period. When examining fall prevention program delivery by program type, AMOB was the most widely disseminated (30,132 participants; 2505 workshops; and, 345 counties), followed by SO (6388 participants; 524 workshops; and, 99 counties), TJQMBB (5332 participants; 410 workshops; and, 39 counties), and TCA (3184 participants, 202 workshops, and 47 counties). In the U.S., there are 3221 counties [43]: 1236 metro counties, 1034 non-metro adjacent counties, and 951 non-metro non-adjacent counties. Collectively, fall prevention program workshops were delivered in 551 counties (17.1%), one or more times, from August 2014 to July 2017. Of the counties where a workshop was conducted, 43.6% were delivered in non-metro areas. Workshops were delivered in 311 of the 1236 metro counties (25.2%), 154 of the 1034 non-metro adjacent counties (14.9%), and 86 of the 951 non-metro non-adjacent counties (9.0%). In addition, there was geospatial clustering of programs throughout the U.S., especially in the Midwest, with smaller/narrower clusters in the south (Texas), the southeast, and the northeast. There were also major gaps in program delivery throughout the nation outside of these clusters, and the majority of the U.S. was without any program delivery.

Table 2 describes the delivery of fall prevention programs by delivery site-type and rurality, in terms of the number of participants enrolled, the number of workshops delivered, and the number of counties reached. Overall, the five most prevalent delivery site types were senior centers (11,989 participants; 963 workshops; and, 170 counties), residential facilities (8933 participants; 731 workshops; and, 84 counties), healthcare organizations (6087 participants; 629 workshops; and, 71 counties), faith-based organizations (3874 participants; 298 workshops; and, 49 counties), and recreational organizations (3111 participants; 228 workshops; and, 31 counties). In non-metro non-adjacent areas, the five most prevalent delivery sites were senior centers, residential facilities, healthcare organizations, community centers, and faith-based organizations.

Table 3 describes the participant (from self-report surveys), delivery site location (from ZCTA of the delivery site), and workshop characteristics (from administrative records) by rurality. Overall, the average participant age was 76.01 (±9.45) years. The majority of participants was female (80.5%) and white (73.4%). Small proportions of participants were African American (6.9%) and Hispanic (5.9%). About 11% of participants had less than a high school education, with 44.9% reporting a high school diploma, General Education Diploma (GED), or vocational school and 44.1% reporting a college degree or higher. The four most commonly self-reported chronic conditions were arthritis (42.0%), heart disease (19.7%), diabetes (16.0%), and depression/anxiety (10.6%). These proportions remained consistent across rurality, although smaller proportions of participants in rural counties were African American or Hispanic (relative to those who participated in metro counties). 

On average, workshops were delivered in areas with median household incomes of $55,861 (±$21,668.27); 14.06% (±9.52%) of residents living in poverty; 79.26% (±19.44%) of residents being white; 9.88% (±15.31%) of residents being African American; 12.99% (±18.00%) of residents being Hispanic; and, 12.97% (±9.18%) of participants having less than a high school education. When compared to workshops that were delivered in metro areas, workshops delivered in non-metro non-adjacent areas had lower median household incomes (i.e., average income of $58,346.23 compared to $45,857.62), had more white residents (i.e., 76.84% as compared to 89.87%), had fewer African American residents (i.e., 11.25% compared to 2.73%), and had fewer Hispanic residents (i.e., 14.79% compared to 7.04%). On average, fall prevention program workshops enrolled 14.71 (±7.87) participants. On average, participants attended 70.64% (±29.73%) of workshop sessions, and these rates were comparable across rurality.

## 4. Discussion

This study provides an overview of a national dissemination of eight evidence-based fall prevention programs. The majority of participants were reached by workshops that were delivered in metro areas (about 80%), which indicates that fall prevention programming requires additional efforts to expand dissemination in rural areas. The majority of U.S. counties lacked access to fall prevention programs, which is consistent with past studies investigating the national delivery of multiple evidence-based programs (for falls, disease self-management, and physical activity) [19,26]. That said, these programs are reaching multiple areas where millions of older adults reside (in fact, the largest proportion of older adults live in metro areas, especially those ages 85 years and older and of the greatest fall-related risk [46]). While three programs (i.e., AMOB, SO, and TJQMBB) reached over 91% of participants in this initiative, this national effort utilized a diverse set of programs, with varying delivery formats and requirements, because a one-size-fits-all approach cannot adequately meet the diverse needs of older adults. Further, findings highlight the embedment of these programs within diverse delivery site types, which confirms the findings from previous studies [29,44] and is important to bridge the aging services network, public health sector, and healthcare system. To increase older adults’ access to and utilization of evidence-based fall prevention programs, interventions must be delivered where older adults feel comfortable and regularly congregate. Often, this may require additional and creative efforts to address transportation issues among older adults, which is more challenging in rural areas [14,47,48].

While the delivery site types most frequently hosting fall prevention programs were similar in metro and non-metro areas, there were small differences in the leading five site types. These findings highlight the need to diversify the delivery infrastructure for fall prevention programs to adequately serve older adults in rural areas. This will require substantially expanding the delivery infrastructure through leader training and organizational recruitment (e.g., the use of faith-based organizations and community centers in rural areas). Further, ongoing efforts are needed to offer sustainable technical assistance and to develop scalable clinical-community referral systems to increase fall prevention program participation among rural-dwelling older adults [49]. Such efforts can be used to inform clinicians about the benefits of evidence-based programming, their availability within local communities (i.e., where and when), and referral processes for enrollment. Such efforts can also educate older adults during the intervention about the importance of communicating with their clinicians about their participation, which can enhance and complement the care received. Further, clinical-community partnerships can yield alternative and innovative financial models to support the delivery and expansion of evidence-based fall prevention across the country. These efforts can expand the reach and adoption of fall prevention programs to serve older adults at risk for falls in rural areas.

While capable of serving rural areas, the reach to non-metro counties was modest, especially relative to other more established evidence-based programs (e.g., CDSME) [26,29]. As with any grand-scale dissemination of programing, time is needed to build a national delivery infrastructure that is capable of reaching all areas of the country. Because programs are delivered in locations at the discretion of each grantee, interventions are often initially offered where convenient and in areas with higher population densities. However, as time passes, programming ultimately reach more and more rural areas. As seen in this study, these fall prevention efforts are gaining traction and receiving ongoing support from ACL. In fact, in an effort to most efficiently nurture the delivery infrastructure, ACL developed two funding tracks in 2018 [31]: (a) capacity-building grants to introduce and deliver programs in underserved geographic areas and/or populations; and, (b) sustainable systems grants to significantly increase program participation and implement innovative funding arrangements with sustainability partners to support programs beyond the grant period.

As an ongoing initiative, new evidence-based fall prevention programs are being introduced and vetted through a multi-tiered and rigorous process, expanding the highest-tier program list [21]. At the bequest of the ACL, the National Council on Aging (NCOA), in partnership with the Evidence-Based Leadership Council (EBLC), established a process for reviewing new and existing community-based programs to determine whether they meet the criteria established by ACL for evidence-based programs funded through the Older Americans Act Title III-D. Independent peer reviewers with expertise in intervention methodology and evaluation assess the program’s research evidence about the program’s effectiveness on health-related or fall prevention outcomes and appraise the quality of dissemination products (e.g., training, materials, and technical assistance) to support the program’s replication into other communities or settings. Programs meeting the basic requirements are added to the highest-tier program list [21]. The process will help to introduce more fall prevention programs into the field that may provide greater options of varying costs, training requirements, and appeal for diverse populations. Expanding these options may make it easier for organizations to increase the number of programs and participants reached in rural regions.

One possible cause of less than optimal dissemination of fall prevention programming in rural areas is the convenience of serving larger communities and meeting grant requirements where older adult populations are abundant. This is also evidenced by our findings that fall prevention program participants were less racially/ethnically diverse relative to the county demographics where programs were delivered. Thus, participation in these programs may have been more associated with participant characteristics than merely the rurality of where the intervention was offered. This emphasizes the need for purposive recruitment strategies to engage racial/ethnic minorities and additional investigations to assess the drivers of race/ethnicity-based participation in fall prevention programs.

Grantees must intentionally develop delivery infrastructures in rural communities and engage with local partners to increase program availability without imposing long drive times for workshop facilitators or participants. As such, opportunities exist to translate programs in terms of content and delivery modality, which can facilitate the expansion of services to new audiences (e.g., homebound populations) in geospatially remote and rural areas. Examples of three primary methods of translation to reach new populations are described briefly here: (a) from face-to-face to virtual delivery; (b) from professionals to lay facilitators; and, (c) from existing content to other cultural- and needs-based tailoring.

Internet-based translations can be effective and they can overcome geospatial barriers if modifications adhere to the critical elements of the original intervention and are made accessible and affordable to underserved areas. While interventions like OEP have been successfully translated for online delivery (i.e., Stand Tall) [50], additional programs, such as AMOB and SO, are currently undergoing virtual translation. Further, SAIL and OEP have online training available, which addressed the cost- and travel-related concerns that are associated with getting trained face-to-face. While professionally-led interventions can cover more advanced and technical content with participants, they are often expensive and prohibitive for grand-scale dissemination (especially in rural areas). Fall prevention programs, such as AMOB and SAIL, can be facilitated by trained lay leaders. OEP has been effectively translated for group-based delivery (with oversight from a professional) to reduce delivery costs and enhance dissemination [51,52]. Often, cultural tailoring is necessary to improve the appropriateness and applicability of interventions for different audiences. While this may include translating interventions into languages other than English, cultural adaptations are more involved processes and they can include modifications to content and format. For example, interventions such as AMOB and SO are undergoing translation for the visual and hearing impaired. Additionally, as an example, the NCOA and ACL are leading an American Indian/Alaskan Native/Native Hawaiian Evidence-Based Program Advisory Council to increase tribal community access to and participation in health promotion, disease prevention, and fall prevention programs, which includes culturally adaptable evidence-based programs as well as initiatives that are developed by native communities. 

### Limitations

This study is not without limitations. The analyses performed were descriptive in nature using pooled data, and as such provides only a snapshot or cross-section of the overall reach of these fall prevention programs. Workshops were clustered/regional in nature and some areas are more likely to offer a particular program type over another. This clustering is an artifact that data were collected from grantees, and grants were not evenly distributed across all states. Further, grantees were able to select locations for program delivery, which were often in more metro and population-dense areas. It should also be noted that the data used in these analyses were from fall prevention programs that were delivered by ACL-funded grantees and do not represent all fall prevention programs delivered nationwide. While non-grantees are able and encouraged to use the ACL-sponsored data repository, this is only required of official ACL grantees. The proportion of participant characteristics (e.g., sex, race, ethnicity) did not always represent the ZIP Code estimates (e.g., less diversity in self-reported measures, less heterogeneity existing among older populations compared to total county estimates), which may be indicative of convenience samples and the locations where the programs were offered. This discordance may limit the generalizability of findings. Additionally, while this study reported the number of participants who enrolled in fall prevention interventions, it did not document the success of actual recruitment efforts (e.g., the proportion of participants who attended workshops relative to those invited to attend). The delivery of evidence-based programs is dependent on several factors (e.g., available resources, infrastructure); therefore, the decision to deliver a fall prevention program may be made with very different goals, preferences, available funding, target populations, and partnerships. For example, the decision and/or ability for a particular aging services organization or stakeholder to provide services to rural-residing individuals in one location may be extremely different than such decisions/abilities in another area. Further research is needed to identify the factors that are associated with program delivery decisions by grantees and their impact on participant recruitment in rural areas. While data pertaining to self-reported falls and other health indicators were collected from participants as part of this initiative, evaluating the effectiveness of these interventions is outside the scope of this descriptive geospatial study and an area for future research. Despite these limitations, this study is among the first examinations of a national dissemination of multiple, diverse evidence-based fall prevention programs.

## 5. Conclusions

Ongoing efforts such as this are needed to assess the delivery infrastructure and geospatial reach and adoption of evidence-based fall prevention programs. Findings from such initiatives can inform key stakeholders, including those affecting policy (e.g., federal funding organizations, state representatives), about successes and challenges to fuel informed decisions about funding priorities, and areas/regions of greatest need. Interventions are needed to prevent falls and mitigate fall-related consequences among individuals, their families, and greater society. Supporting the delivery of a diverse cadre of evidence-based fall prevention interventions ensures the diverse needs and programming preferences of older adults can be met. While there is no one-size-fits-all model for fall prevention programming, the availability of multiple effective interventions are made available to older adults with differing levels of risk across rurality. Given that rural areas have limited healthcare resources relative to metro areas, evidence-based fall prevention programming can complement traditional medical and clinical services by being embedded in an array of community-based settings.

## Figures and Tables

**Table 1:** Fall Prevention Program Workshop Types by Rurality.

Program Type	PARTICIPANTS				WORKSHOPS (COUNTIES)			
	Metro	Non-Metro: Adjacent	Non-Metro: Non-Adjacent	Total	Metro	Non-Metro: Adjacent	Non-Metro: Non-Adjacent	Total
A Matter of Balance	25,217	3176	1739	30,132	2057 (204)	289 (91)	159 (50)	2505 (345)
Stepping On	4820	1133	435	6388	388 (49)	96 (35)	40 (15)	524 (99)
Tai Ji Quan	4095	757	480	5332	295 (17)	65 (12)	50 (10)	410 (39)
Tai Chi for Arthritis	2439	492	253	3184	157 (30)	30 (11)	15 (6)	202 (47)
FallScape	26	124	112	262	3 (2)	3 (3)	7 (5)	13 (10)
SAIL	242	0	0	242	14 (3)	0 (0)	0 (0)	14 (3)
Stay Safe, Stay Active	6	138	0	144	1 (0)	2 (1)	0 (0)	3 (1)
Otago Exercise Program	127	1	0	128	83 (6)	1 (1)	0 (0)	84 (7)
TOTAL	36,972	5821	3019	45,812	2998 (311)	486 (154)	271 (86)	3755 (551)
(0) = no unique county served with this program (i.e., program may have been offered in a county where a different program was served)								

**Table 2:** Delivery Site Types by Rurality.

Delivery Site Type	PARTICIPANTS				WORKSHOPS (COUNTIES)			
	Metro	Non-Metro: Adjacent	Non-Metro: Non-Adjacent	Total	Metro	Non-Metro: Adjacent	Non-Metro: Non-Adjacent	Total
Senior Center	9618	1564	807	11,989	739 (94)	129 (49)	68 (27)	936 (170)
Residential Facility	7248	1122	563	8933	582 (46)	98 (23)	51 (15)	731 (84)
Healthcare Organization	4833	763	491	6087	507 (41)	72 (17)	50 (13)	629 (71)
Faith-Based Organization	2992	632	250	3874	229 (23)	50 (18)	19 (8)	298 (49)
Recreational Organization	2877	158	76	3111	206 (24)	13 (5)	9 (2)	228 (31)
Community Center	2148	479	258	2885	168 (16)	37 (8)	19 (3)	224 (27)
Other	2179	396	234	2809	176 (27)	30 (14)	22 (8)	228 (49)
Multi-Purpose/Social Service Organization	1376	89	62	1527	106 (9)	9 (5)	7 (3)	122 (17)
Library	1215	80	77	1372	89 (6)	7 (1)	7 (0)	103 (7)
Area Agency on Aging	846	111	108	1065	69 (13)	13 (7)	10 (5)	92 (25)
Municipal Government	491	108	8	607	40 (1)	9 (2)	1 (0)	50 (3)
County Health Department	416	136	12	564	31 (7)	13 (2)	1 (0)	45 (9)
Educational Institution	433	35	56	524	30 (2)	3 (1)	5 (1)	38 (4)
Tribal Center	11	148	17	176	2 (0)	3 (2)	2 (1)	7 (3)
State Unit on Aging	171	0	0	171	14 (2)	0 (0)	0 (0)	14 (2)
Workplace	102	0	0	102	8 (0)	0 (0)	0 (0)	8 (0)
State Health Department	16	0	0	16	2 (0)	0 (0)	0 (0)	2 (0)
TOTAL	36,972	5821	3019	45,812	2998 (311)	486 (154)	271 (86)	3755 (551)
(0) = no unique county served by this delivery site type (i.e., program may have been offered in a county where a different program was served)								

**Table 3:** Participant, Delivery Site Location, and Workshop Characteristics by Rurality.

Participant Characteristics	Total	Metro	Non-Metro: Adjacent	Non-Metro: Non-Adjacent
Age	76.01 (±9.45)	75.87 (±9.41)	76.42 (±9.47)	76.81 (±9.78)
Proportion of Female Participants	80.5%	80.3%	82.4%	79.9%
Proportion of White Participants	73.4%	71.1%	83.7%	82.0%
Proportion of African American Participants	6.9%	7.8%	3.9%	1.5%
Proportion of Hispanic Participants	5.9%	6.8%	2.0%	2.6%
Proportion with Less Than High School	11.1%	11.0%	11.6%	10.4%
Proportion with High School/GED/Vocational	44.9%	43.0%	53.5%	49.4%
Proportion with College Degree or Higher	44.1%	46.0%	34.8%	40.2%
Proportion with Arthritis	42.0%	41.2%	45.7%	44.7%
Proportion with Heart Disease	19.7%	19.1%	22.1%	22.6%
Proportion with Diabetes	16.0%	15.8%	17.0%	16.5%
Proportion with Depression or Anxiety	10.6%	10.6%	11.2%	9.9%
Delivery Site Location Characteristics *				
Median Household Income *	$55,861.66 (±21,668.27)	$58,346.23 (±22,932.93)	$45,313.00 (±9922.84)	$45,857.62 (±10,303.06)
Percent Living Over Poverty Line *	14.06 (±9.52)	14.01 (±10.06)	14.42 (±7.04)	13.96 (±6.32)
Percent White *	79.26 (±19.44)	76.84 (±19.34)	89.11 (±17.38)	89.87 (±14.11)
Percent African American *	9.88 (±15.31)	11.25 (±15.96)	4.88 (±11.46)	2.73 (±7.83)
Percent Hispanic *	12.99 (±18.00)	14.79 (±18.83)	4.61 (±8.30)	7.04 (±15.18)
Percent Less than High School Education *	12.97 (±9.18)	12.94 (±9.64)	13.31 (±7.15)	12.66 (±6.43)
**Workshop Characteristics**				
Number of Participants Enrolled in Workshops	14.71 (±7.87)	14.69 (±6.99)	15.64 (±12.62)	13.12 (±5.43)
Proportion of Sessions Attended	70.64 (±29.73)	70.81 (±29.49)	70.19 (±30.39)	69.41 (±31.45)

* Indicates statistic from the ZCTA of the delivery site location.

## References

[1] Bergen G. (2016). Falls and fall injuries among adults aged ≥65 years—United States, 2014. MMWR.

[2] Carroll N.V., Slattum P.W., Cox F.M. (2005). The cost of falls among the community-dwelling elderly. J. Manag. Care Pharm..

[3] Davis J.C., Robertson M.C., Ashe M.C., Liu-Ambrose T., Khan K.M., Marra C.A. (2010). International comparison of cost of falls in older adults living in the community: A systematic review. Osteoporose. Int..

[4] Towne S.D., Ory M.G., Smith M.L. (2014). Cost of fall-related hospitalizations among older adults: Environmental comparison from the 2011 Texas Hospital Inpatient Discharge Data. Popul. Health Manag..

[5] Centers for Disease Control and Prevention (2017). Take a Stand on Falls: What Can Older Adults Do to Prevent Falls?. https://www.cdc.gov/features/older-adult-falls/index.html.

[6] Towne S.D., Fair K., Smith M.L., Dowdy D.M., Ahn S., Nwaiwu O., Ory M.G. (2017). Multilevel Comparisons of Hospital Discharge among Older Adults with a Fall-Related Hospitalization. Health Serv. Res..

[7] United States Census Bureau (2018). Older People Projected to Outnumber Children for First Time in U.S. History. https://www.census.gov/newsroom/press-releases/2018/cb18-41-population-projections.html.

[8] Ory M.G., Smith M.L. (2015). Evidence-Based Programming for Older Adults.

[9] Stevens J.A. (2016). A CDC Compendium of Effective Fall Interventions: What Works for Community-Dwelling Older Adults.

[10] Fabre J.M., Ellis R., Kosma M., Wood R.H. (2010). Falls Risk Factors and a Compendium of Falls Risk Screening Instruments. J. Geriatr. Phys. Ther..

[11] Rubenstein L.Z. (2006). Falls in older people: epidemiology, risk factors and strategies for prevention. Age Ageing.

[12] Coben J.H. (2009). Rural-urban differences in injury hospitalizations in the U.S., 2004. Am. J. Prev. Med..

[13] Yiannakoulias N., Rowe B.H., Svenson L.W., Schopflocher D.P., Kelly K., Voaklander D.C. (2003). Zones of prevention: The geography of fall injuries in the elderly. Soc. Sci. Med..

[14] Goins R.T., Williams K.A., Carter M.W., Spencer S.M., Solovieva T. (2005). Perceived barriers to health care access among rural older adults: A qualitative study. J. Rural Health.

[15] Nicholson N.R. (2012). A review of social isolation: An important but underassessed condition in older adults. J. Prim. Prev..

[16] Towne S.D., Probst J.C., Smith M.L., Salinas M., Ory M.G. (2016). Rural Health and Aging: Global Perspectives Encyclopedia of Geropsychology.

[17] Weber V., White A., McIlvried R. (2008). An electronic medical record (EMR)-based intervention to reduce polypharmacy and falls in an ambulatory rural elderly population. J. Gen. Int. Med..

[18] Chan L., Hart L.G., Goodman D.C. (2006). Geographic access to health care for rural Medicare beneficiaries. J. Rural Health.

[19] Goins R.T., Krout J.A. (2006). Service Delivery to Rural Older Adults: Research, Policy, and Practice.

[20] Towne S.D., Smith M.L., Pulczinski J.C., Lee C., Ory M.G. Rural Healthy People 2020: A Companion Document to Healthy People 2020. https://srhrc.tamhsc.edu/rhp2020/rhp2020-v1-download.html.

[21] National Council on Aging (2018). Highest Tier Evidence-Based Health Promotion/Disease Prevention Programs. https://www.ncoa.org/wp-content/uploads/Title-IIID-Highest-Tier-EBPs-June-28-2018.pdf.

[22] Frieson C.W., Tan M.P., Ory M.G., Smith M.L. (2018). Evidence-based practices to reduce falls and fall-related injuries among older adults. Front. Public Health.

[23] Yates S.M., Dunnagan T.A. (2001). Evaluating the effectiveness of a home-based fall risk reduction program for rural community-dwelling older adults. J. Gerontol. Ser. A.

[24] Jeon M.Y., Jeong H., Petrofsky J., Lee H., Yim J. (2014). Effects of a randomized controlled recurrent fall prevention program on risk factors for falls in frail elderly living at home in rural communities. Med. Sci. Monit..

[25] Smith M.L., Ahn S., Sharkey J.R., Horel S., Mier N., Ory M.G. (2012). Successful falls prevention programming for older adults in Texas: Rural-urban variations. J. Appl. Gerontol..

[26] Towne S.D., Smith M.L., Ahn S., Altpeter M., Belza B., Kulinski K.P., Ory M.G. (2015). National dissemination of multiple evidence-based disease prevention programs: Reach to vulnerable older adults. Front. Public Health.

[27] Smith M.L., Quinn C., Gipson R., Wilson A.D., Ory M.G. (2011). Serving rural communities for falls prevention: The dissemination of A Matter of Balance in the Brazos Valley region of Texas. Texas Public Health Assoc. J..

[28] Smith M.L., Towne S.D., Motlagh A.S., Smith D., Boolani A., Horel S.A., Ory M.G. (2017). Programs and place: Risk and asset mapping for fall prevention. Front. Public Health.

[29] Smith M.L., Towne S.D., Herrera-Venson A., Cameron K.A., Kulinski K.P., Lorig K., Horel S.A., Ory M.G. (2017). Dissemination of Chronic Disease Self-Management Education (CDSME) Programs in the United States: Intervention delivery by rurality. Int. J. Environ. Res. Public Health.

[30] Administration for Community Living (2017). Empowering Older Adults and Adults with Disabilities through Chronic Disease Self-Management Education (CDSME) Programs Financed by 2017 Prevention and Public Health Funds (HHS-2017-ACL-AOA-CSSG-0207).

[31] Administration for Community Living (2017). Evidence-Based Falls Prevention Programs Financed Solely by 2017 Prevention and Health Funds (HHS-2017-ACL-AOA-FPSG-0206).

[32] Kulinski K.P., Boutaugh M.L., Smith M.L., Ory M.G., Lorig K. (2015). Setting the stage: Measure selection, coordination, and data collection for a national self-management initiative. Front. Public Health.

[33] U.S. Department of Agriculture (2017). Rural-Urban Continuum Codes. https://www.ers.usda.gov/data-products/rural-urban-continuum-codes.aspx.

[34] Hall S.A., Kaufman J.S., Ricketts T.C. (2006). Defining urban and rural areas in U.S. epidemiologic studies. J. Urban Health.

[35] Minore B., Hill M.E., Pugliese I., Gauld T. (2008). Rurality Literature Review.

[36] Towne S.D., Smith M.L., Ory M.G. (2014). Geographic variations in access and utilization of cancer screening services: Examining disparities among American Indian and Alaska Native Elders. Int. J. Health Geogr..

[37] Healy T.C., Peng C., Haynes M.S., McMahon E.M., Botler J.L., Gross L. (2008). The feasibility and effectiveness of translating a matter of balance into a volunteer lay leader model. J. Appl. Gerontol..

[38] Clemson L., Cumming R.G., Kendig H., Swann M., Heard R., Taylor K. (2004). The effectiveness of a community-based program for reducing the incidence of falls in the elderly: A randomized trial. J. Am. Geriatr. Soc..

[39] Li F. (2014). Transforming traditional Tai Ji Quan techniques into integrative movement therapy—Tai Ji Quan: Moving for Better Balance. J. Sport Health Sci..

[40] Callahan L.F., Cleveland R.J., Altpeter M., Hackney B. (2016). Evaluation of Tai Chi program effectiveness for people with arthritis in the community: A randomized controlled trial. J. Aging Phys. Act..

[41] Renfro M., Maring J., Bainbridge D., Blair M. (2016). Fall risk among older adult high-risk populations: A review of current screening and assessment tools. Curr. Geriatr. Rep..

[42] Barnett A., Smith B., Lord S., Williams M., Baumand A. (2003). Community-based group exercise improves balance and reduces falls in at-risk older people: A randomized controlled trial. Age Ageing.

[43] Shubert T.E., Smith M.L., Jiang L., Ory M.G. (2018). Disseminating the Otago exercise program in the United States: Perceived and actual physical performance improvements from participants. J. Appl. Gerontol..

[44] Smith M.L., Ory M.G., Ahn S., Belza B., Mingo C.A., Towne S.D., Altpeter M. (2015). Reaching diverse participants utilizing a diverse delivery infrastructure: A replication study. Front. Public Health.

[45] Smith M.L., Belza B., Altpeter M., Ahn S., Dickerson J.B., Ory M.G., Maddock J. (2012). Disseminating an evidence-based disease self-management program for older Americans: Implications for diversifying participant reach through delivery site adoption. Public Health: Social and Behavioral Health.

[46] Rural Health Information Hub (2018). Demographic Changes and Aging Population, Rural Aging in Place Toolkit. https://www.ruralhealthinfo.org/toolkits/aging/1/demographics.

[47] Arcury T.A., Preisser J.S., Gesler W.M., Powers J.M. (2005). Access to transportation and health care utilization in a rural region. J. Rural Health.

[48] Mattson J. (2011). Transportation, distance, and health care utilization for older adults in rural and small urban areas. Transp. Res. Rec..

[49] Shubert T.E., Smith M.L., Schneider E.C., Wilson A.D., Ory M.G. (2016). Commentary: Public health system perspective on implementation of evidence-based fall prevention strategies for older adults. Front. Public Health.

[50] Shubert T.E., Chokshi A., Mendes V.M., Grier S., Buchanan H., Basnett J., Smith M.L. (2018). Stand Tall: A Virtual Translation of the Otago Exercise Program. J. Geriatr. Phys. Ther..

[51] Shubert T.E., Ory M.G., Jiang L., Smith M.L. (2017). The Otago Exercise Program in the United States: A comparison of two implementation models. Phys. Ther. J..

[52] Shubert T.E., Goto L.S., Smith M.L., Jiang L., Rudman H., Ory M.G. (2017). The Otago Exercise Program: Innovative delivery models to maximize outcomes for high risk, homebound older adults. Front. Public Health.

